# Fetal alcohol spectrum disorder resources for educators: A scoping review

**DOI:** 10.1002/hpja.574

**Published:** 2022-01-26

**Authors:** Briana Lees, Julia Riches, Louise Mewton, Elizabeth J. Elliott, Steve Allsop, Nicola Newton, Sue Thomas, Lauren J. Rice, Smriti Nepal, Maree Teesson, Lexine A. Stapinski

**Affiliations:** ^1^ 4334 The Matilda Centre for Research in Mental Health and Substance Use The University of Sydney Camperdown New South Wales Australia; ^2^ Centre for Healthy Brain Ageing University of New South Wales Sydney New South Wales Australia; ^3^ 4334 Faculty of Medicine and Health Specialty of Child and Adolescent Health The University of Sydney Sydney New South Wales Australia; ^4^ 90098 The Sydney Children’s Hospitals Network Sydney New South Wales Australia; ^5^ National Drug Research Institute Curtin University Perth Western Australia Australia; ^6^ Marulu Unit Marninwarntikura Women’s Resource Centre Fitzroy Crossing Western Australia Australia; ^7^ 4334 The Brain and Mind Centre The University of Sydney Camperdown New South Wales Australia

**Keywords:** education, fetal alcohol spectrum disorder, neurodevelopmental disorder, prenatal alcohol exposure, review, teachers

## Abstract

Children with foetal alcohol spectrum disorder (FASD) can experience neurodevelopmental, physical, psychological and behavioural impairments that can result in a disrupted school experience. However, educators often have limited knowledge or experience in the identification and support of students with FASD, and there is a critical need for effective tools and resources to ensure students with FASD are supported in their ongoing learning and development. This scoping review aimed to identify and evaluate publicly available educator resources that aid in the identification, and support of students with FASD in primary/elementary school. In addition, educators and FASD experts were consulted to obtain feedback on currently available resources, and key issues and priorities for FASD resources. In total, 124 resources were identified by searching peer‐reviewed and grey literature databases, app stores, podcast services and contacting FASD experts. Information was found on identification (23 resources) and support of students with FASD (119 resources). No resources provided information on the referral. Most resources were average (40%) to good (33%) quality, as measured by a composite tool based on adaptions of the NHMRC FORM Framework and iCAHE Guideline Quality Checklist. A minority of resources had been formally evaluated (7%). Review findings and consultations with experts and educators indicate a critical need for referral guides, evidence‐based short‐format resources and centralised access for school communities to high‐quality resources. Taken together, this study has identified key areas for future resource development and research to better support primary school students with FASD.

## 1 INTRODUCTION

Foetal alcohol spectrum disorder (FASD) is a diagnostic term that describes individuals affected by prenatal alcohol exposure.^1^ Primary consequences of FASD include neurodevelopmental, physical, psychological and behavioural impairments.^2^ These difficulties can contribute to secondary outcomes, including a disrupted school experience and impacted academic achievement.^3^, ^4^

School educators often have limited knowledge or experience in the identification and support of students with FASD.^5^ Effective resources and tools for educators are crucial to ensure that such students are supported in their ongoing learning, development and school participation. Previous reviews have synthesised tools for FASD identification, support and intervention resources, often developed for caregivers and health professionals. Specifically, reviews have summarised Canadian screening tools for FASD,^6^ and international health, behavioural and education interventions for individuals with FASD.^7^, ^8^, ^9^, ^10^ Although the identified tools and interventions are important, educators require resources that are tailored for the school setting and outline strategies for supporting learning within the classroom. To date, no review has comprehensively collated and assessed FASD‐related resources designed to equip educators to effectively identify and support students with FASD.

A scoping review was conducted to meet this need. The review aimed to identify and evaluate publicly available resources for educators that aid in identifying and providing learning support for primary school‐aged students with FASD. The review focused specifically on resources for primary school educators because early identification and intervention are associated with reduced risk of long‐term adverse outcomes.^2^, ^11^, ^12^

## 2 METHODS

Ethical approval was provided by the University of XXX Human Research Ethics Committee (2020/825). The pre‐registered review protocol^13^ was informed by frameworks developed by Arksey and O’Malley,^14^ Levac et al^15^ and the Joanna Briggs Institute^16^ which organise the review process in six stages. Details of each stage are available in the published protocol^13^ and briefly described below and in the Supplement.

### 2.1 Stage 1: Research questions

The primary research question was “what resources and/or guidelines are available for educators that (a) enhance identification, assessment, and referral of students with developmental, learning, social and behavioural problems consistent with FASD and (b) aid support or accommodation of FASD‐related symptoms and behaviours in a school setting?”. Secondary research questions included “what is the quality of existing resources?” and “what are the effective components of resources designed to improve the support or accommodation of developmental, learning, social and behavioural problems associated with FASD?”.

### 2.2 Stage 2: Identifying resources

Resources were identified by searching nine peer‐reviewed databases; 11 grey literature websites; two app stores; two podcast streaming services; contacting 15 FASD and education sector experts. The search terms and strategies are provided in Table S1. Resources were defined as any existing literature, such as, but not limited to, primary research studies, reviews, guides, policies, books, student‐focused programs, professional development tools, factsheets, videos, podcasts and apps. Resources were included in the review if they were: (1) relevant for primary/elementary school educators; (2) designed to build capacity amongst educators in identifying students with problems consistent with FASD, or supporting students with FASD; (3) currently publicly available in English, including free and fee‐based resources. Student‐focused programs described in primary research studies were only included if the program was currently publicly available.

### 2.3 Stage 3: Resource selection

A three‐phase screening and selection process was undertaken. First, the titles/abstracts/ descriptions of resources were screened for eligibility by one co‐author, with 20% of retrieved resources additionally screened by a second co‐author. Inter‐rater reliability was high (96% agreement, kappa = 0.787). Second, full‐text versions and data sources of potentially relevant resources were assessed by two co‐authors to determine whether the resource would be included in the review (93% agreement, kappa = 0.851). Finally, reference lists of identified materials were screened to identify other relevant resources.

### 2.4 Stages 4‐5: Collating results and quality appraisal

Resource characteristics were tabulated to summarise relevant data (Table 1). The quality of included resources was assessed using a composite of two tools: an adapted version of the National Health and Medical Research Council (NHMRC) FORM framework^17^ and the iCAHE Guideline Quality Checklist.^18^ One co‐author evaluated each resource for their evidence base, impact and utility, generalisability, availability, currency, ease of use, credibility and applicability (Table S2). Applicability was considered for the Australian context because the scoping review was intended to inform an Australian FASD education initiative. A second co‐author appraised 20% of included resources to ensure consistent evaluation (90% agreement). Each component received a score ranging from poor to excellent. Based on individual component scores, an overall weighted score for each resource was calculated. Evidence base (*3), impact and utility (*2) and currency scores (*2) were given a higher weighting than all other quality components (*1) (Table 2).

**TABLE 1:** Characteristics of resources included in the review

	Total (N = 124)		Identification resources (N = 23)		Support resources (N = 119)	
	N	%	N	%	N	%
Resource type						
Screening tools	5	4.0	5	21.7	‐	‐
Text‐based	75	60.5	16	69.6	75	63.0
Longer guides/books	38	(50.7)	14	(87.5)	38	(50.7)
Short factsheets/booklets	22	(29.3)	‐	‐	22	(29.3)
Classroom planning tools	3	(4.0)	‐	‐	3	(4.0)
Theses	2	(2.7)	‐	‐	2	(2.7)
Literature reviews	8	(10.7)	2	(12.5)	8	(10.7)
Primary research only	2	(2.7)	‐	‐	2	(2.7)
Videos	26	21.0	1	4.3	26	21%
Short (<20 min)	22	(84.6)	‐	‐	22	(84.6)
Longer webinars/lectures	4	(15.4)	1	(100.0)	4	(15.4)
Podcast episodes	10	8.1	‐	‐	10	8.4
Games for students	2	1.6	‐	‐	2	1.7
Professional development	4	3.2	1	4.3	4	3.4
Programs/interventions	2	1.6	‐	‐	2	1.7
Apps	‐	‐	‐	‐	‐	‐
Peer‐reviewed	27	21.8	9	39.1	25	21.0
Resource evaluated	8	6.5	3	13.0	5	4.2
Country of origin						
United States	51	41.1	10	43.5	49	41.2
Canada	44	35.5	9	39.1	42	35.3
Australia	12	9.7	2	8.7	11	9.2
United Kingdom	11	8.9	1	4.3	11	9.2
New Zealand	5	4.0	1	4.3	5	4.2
Netherlands	1	0.8	‐	‐	1	0.8
Cost						
Free	110	88.7	19	82.6	105	88.2
Low cost (<$100)	12	9.7	4	17.4	12	10.1
High cost ($≥$100)	2	1.6	‐	‐	2	1.7
Access						
Available online	119	96.0	21	91.3	114	95.8
Hard copy only	5	4.0	2	8.7	5	4.2
Currency						
Developed in previous 10 years	81	65.3	13	56.5	77	64.7
Developed ≥10 years ago	43	34.7	10	43.5	42	35.3

Note

Percentage breakdowns for text‐based and video resource sub‐types are provided in brackets. Due to rounding, percentages do not always equate to 100%.

**TABLE 2:** Overall quality of resources

	N	Excellent (%)	Good (%)	Average (%)	Poor (%)	Very Poor (%)
Identification resources	**23**	**13.0**	**39.1**	**34.8**	**8.7**	**4.3**
Screening tools	5	40.0	—	60.0	—	**—**
Text‐based	16	6.3	50.0	25.0	12.5	6.3
Longer guides/books	14	7.1	37.5	25.0	12.5	6.3
Literature review	2	—	100.0	—	—	—
Videos (webinar)	1	—	100.0	—	—	—
Professional development	1	—	—	100.0	—	—
Support resources	**119**	**2.5**	**34.5**	**39.5**	**8.4**	**15.1**
Text‐based	75	2.7	40.0	38.7	12.0	**6.7**
Longer guides/books	38	2.6	44.7	34.2	13.2	5.3
Short factsheets/booklets	22	0.0	22.7	59.1	9.1	9.1
Classroom planning tools	3	—	—	33.3	33.3	33.3
Thesis	2	50.0	50.0	—	—	—
Literature reviews	8	—	75.0	12.5	12.5	—
Primary research only	2	—	50.0	50.0	—	—
Videos	26	—	15.4	42.3	3.8	38.5
Short videos	22	—	9.1	40.9	4.5	45.5
Webinars / lectures	4	—	50.0	50.0	—	—
Podcasts	10	—	20.0	60.0	20.0	—
Professional development	4	—	75.0	25.0	—	—
Programs	2	50.0	50.0	—	—	—

Note

The bold values indicate the overall quality scores for each resource type (identification and support).

### 2.5 Stage 6: Consultation with key stakeholders

FASD experts, identified via team networks and online searches, completed online surveys to provide feedback on high‐quality resources from each resource type and identified gaps in currently available resources for educators. Additionally, Australian educators, recruited on social media, were consulted via online surveys and asked to provide feedback on FASD resources they have previously accessed barriers to obtaining information about FASD, and resources that they require to support students with FASD. Due to word constraints, these results are provided in the Supplement. The surveys took approximately one hour and included a combination of quantitative and qualitative open‐ended responses. Experts and educators were offered $40 as reimbursement for their time.

## 3 RESULTS

### 3.1 Overview

**FIGURE 1:**
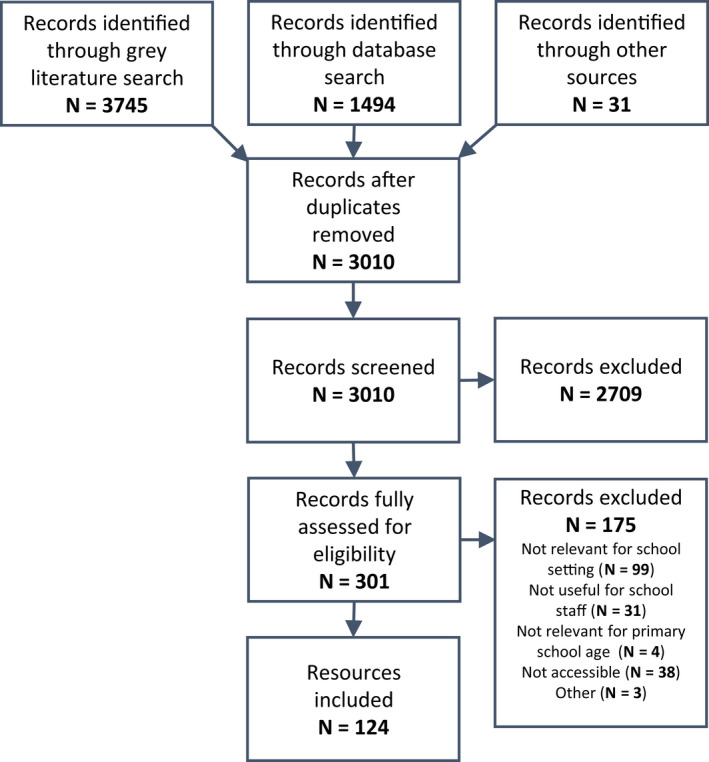
Resources included in the review

Searches were conducted up to February 2021. Of 3010 resources identified through the search strategy and screened, 124 met the criteria and were included in this review (Figure 1). Ninety‐seven were identified in the grey literature, 21 in peer‐reviewed databases and six were identified by FASD experts. All included resources are listed in the Supplement File (Table S3).

Resources were developed in the United States (n = 51;41%), Canada (n = 44;36%), Australia (n = 12;10%), the United Kingdom (n = 11;9%), New Zealand (n = 5;4%) and the Netherlands (n = 1;<1%). Development dates ranged from 1969 to 2021, with 50% developed in the previous eight years (2013‐2021).

Most resources focused on support of FASD‐related symptoms and behaviours (n = 119). A minority focused on identification of FASD indicators (n = 23). Most resources were text‐based (n = 75;61%) and a smaller portion were videos (n = 26;21%), podcast episodes (n = 10;8%), screening tools (n = 5;4%), student‐focused games (n = 2;2%), interventions (n = 2;2%) and professional development resources (n = 4;3%). Notably, 89% of resources identified were free for educators.

### 3.2 Quality of resources

**FIGURE 2:**
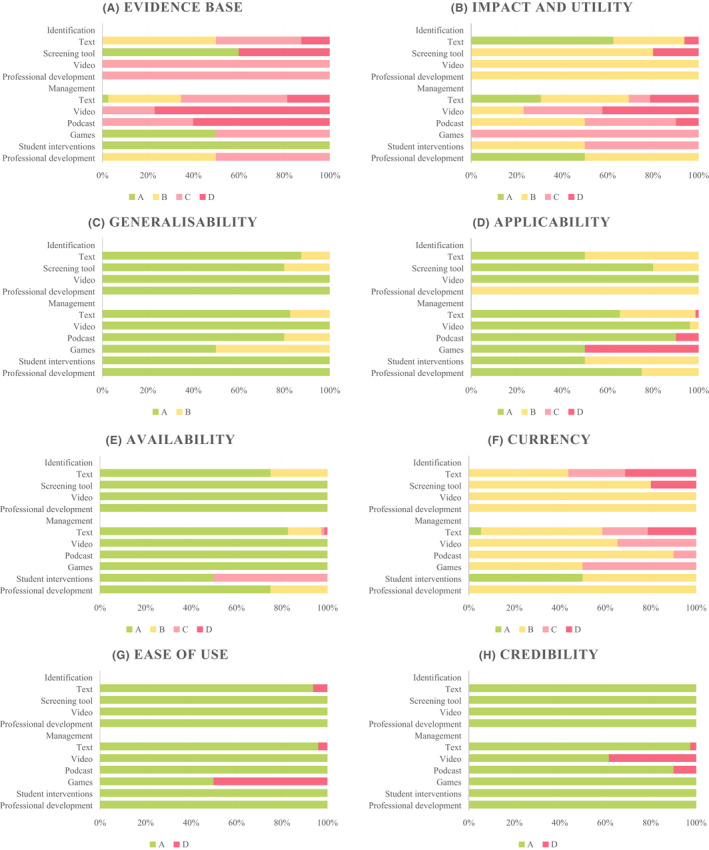
Summary of appraisal scores for each quality component. Note: A = excellent rating; B = good rating; C = satisfactory rating; D = poor rating

The overall quality of resources is summarised in Table 2 and a breakdown of quality components is provided in Figure 2. Most resources were of average (35% of identification resources, 40% of support resources) or good quality (39% of identification resources, 35% of support resources). Notably, just 13% of identification resources and 3% of support resources received an excellent quality score.

There was considerable variation in the quality of the evidence base, currency, and the impact and utility of resources (Figure 2). In terms of the evidence base, 7% of resources were formally evaluated with findings published, 21% were developed based on published findings, 39% were developed based on expert consensus but made no reference to formal evidence or user testing, and 33% were developed based on personal opinion only. Regarding currency, 65% of resources were developed in the previous decade and were considered current (although only 4% were regularly updated), 21% were developed over 10 years ago (not considered current) but did not contain out‐of‐date information, and 14% were out‐dated. For impact and utility, 58% of resources covered more than one issue related to FASD in the school setting, with 20% of these resources covering the issues comprehensively. Finally, the majority of resources received excellent scores for generalisability (ie, relevant to target group; 86%), availability (ie, no associated costs; 88%), ease of use (97%), and credibility (ie, university‐based, government‐funded or other reputable developers; 89%). The majority of resources were directly applicable to the Australian context (74%).

### 3.3 Identification and referral resources

Of the 23 included resources which provide information to aid identification of students with FASD indicators, 16 were text‐based (70%), 5 were screening tools (22%), 1 was a video (4%) and 1 was a professional development resource (4%) (Table 1). No resources included in the review provided guidance on referral pathways. Nine of 23 resources (39%) were peer‐reviewed and three had been formally evaluated (13%). Two peer‐reviewed resources were current (ie, developed in the previous 10 years) and two evaluated resources had findings published in the previous decade. Data on FASD indicators in the school setting were extracted from the current peer‐reviewed and evaluated resources (n = 4), synthesised in Table 3.

**TABLE 3:** FASD indicators and common presentations in the school setting

Domain	Presentation in a school setting
Physical motor skills	Difficulties in hand/eye and total body coordination, eg: Cutting with scissors, colouring or sewing. Letter formation, holding pencil, slow writing pace. Hitting or catching a ball. Tying shoes.
Sensory processing (sensory‐motor integration and processing pace)	Slow processing pace: Require extended period to take in visual and auditory information and may take minutes to respond. Miss or overlook relevant information due to slow rate of sensory intake. Visuo‐spatial difficulties: Misjudging personal boundaries (eg, stand close to other students). Struggles to remember the locations of the classrooms, misplaces items. Difficulties planning the layout of work on a page (eg, writing in the centre of the page instead of on the upper, left). Difficulties in copying information from the board because of visual distractions in the classroom.
Cognition	Cognitive difficulties: Learning difficulties, poor school performance. Poor impulse control and executive functions. Poor common sense, act without thinking. Do not recognise consequences. Low capacity for abstract thinking. Frustrated or resistant to shift to a new task. Struggles to shift strategy for a new type of problem. Repeatedly makes the same errors on a task. Poor judgement and organisation. Struggles to make a plan, identify goals or predict possible outcomes. Frequently asks what comes next. Difficulties synthesising information to form a conclusion.
Language and communication	Deficits in higher‐level receptive and expressive language, resulting in: Problems following instructions or conversations. Comprehension difficulties. Difficulty in finding words to describe their feelings. Misuse and poor social timing of figurative language, jokes and sarcasm. Difficulties reading and responding to nonverbal cues and body language. Talks better than they understand, eg, makes unrelated remarks to the discussion. Storytelling that is hard to follow and lacks a point.
Academic achievement/developmental stage	Common areas of impacted academic achievement: Math Science Vocabulary Arts Earlier developmental level of functioning: Interests and play are young for their age. Prefers younger friends.
Memory	Problems with encoding, storage, and retrieval, eg: Have remembered or done something many times before, but forget on another given day. Educator provides three instructions, and they only remember to do the first part. Needs to be retaught the same thing multiple times.
Self‐regulation/attention deficit/hyperactivity	Difficulty in maintaining focus (easily distracted by visuals/auditory) and self‐regulating when overstimulated or tired, resulting in: Struggles to sit still in class. Difficulties in stopping the action when asked to do so. Can seem defiant when asked to shift to a calm state. Often works in spurts, requiring assistance to complete a task.
Adaptive behaviour	Decreased capacity to develop new social, practical, and conceptual skills that would assist them in daily living.
Secondary behavioural characteristics	Behaviours that can develop over time as a result of a “poor fit” between the person's needs and the school environment: Fatigue. Anxious, depressed, withdrawn. Lonely, isolated, avoidant. Easily frustrated, aggressive, destructive, argumentative. Easily manipulated by others.

Note

These FASD indicators are also indicators of other presentations, such as attention deficit disorder and oppositional defiant disorder.^19^, ^20^, ^21^, ^22^

#### 3.3.1 Text‐based resources

Fourteen guides and books and two peer‐reviewed literature reviews were identified. Three of the guides were peer‐reviewed but were of poor to average quality and were not current. Guides typically provided information on FASD diagnostic criteria and prevalence rates in schools. One peer‐reviewed book chapter and one literature review were current. The book chapter described how the primary characteristics of FASD typically present within the school setting.^19^ The literature review summarised the common characteristics and associated difficulties related to each of nine functional domains used in the Canadian guidelines for FASD diagnosis.^21^ Data from Millar et al^21^ and Coles et al^19^ are synthesised in Table 3.

#### 3.3.2 Screening tools

Of five screening tools identified, two were evaluated in the previous 10 years and were rated as excellent overall quality. The 10‐item *Neurobehavioural Screening Tool* has 62.5% sensitivity for youth with FASD^23^ and was designed to be administered by a psychologist or youth worker within a school or elsewhere.^22^ The 12‐part *FASCETS Neurobehavioural Screening Tool* has adequate psychometric properties^24^ and explores wide‐ranging neurobehavioural characteristics which may assist educators in the referral process and in assessing management and intervention needs.^20^ Indicators from these screeners are synthesised in Table 3.

#### 3.3.3 Videos

One current, good quality 45‐min webinar was identified; however, no shorter videos were found. The Australian *NOFASD Webinar for Teachers and Educators* provides a description of FASD and discusses the learning challenges for students.

#### 3.3.4 Professional development

One professional training resource was identified. The Canadian resource *Supporting Students with Fetal Alcohol Spectrum Disorder* includes three 10‐15min modules that describe typical behavioural patterns of students with FASD.

### 3.4 Support resources

Support resources (n = 119) included text‐based resources (n = 75), videos (n = 26), podcast episodes (n = 10), games (n = 2) and interventions for students (n = 2), and professional development modules (n = 4) (Table S3). No relevant mobile apps were identified.

#### 3.4.1 Text‐based resources

**FIGURE 3:**
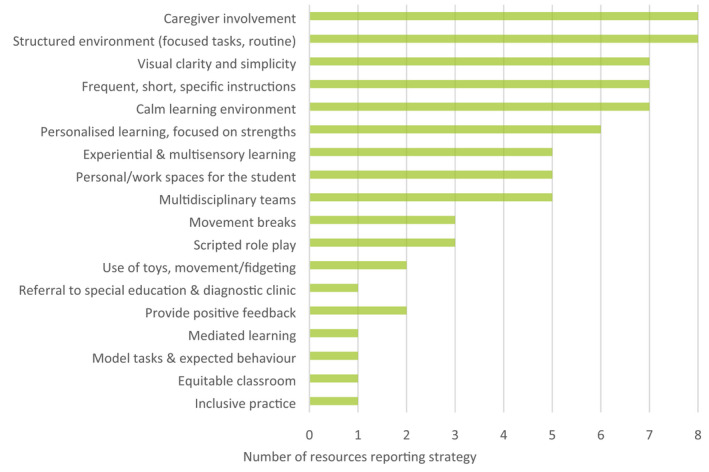
Effective classroom strategies to support students with FASD (total resources = 12)

**FIGURE 4:**
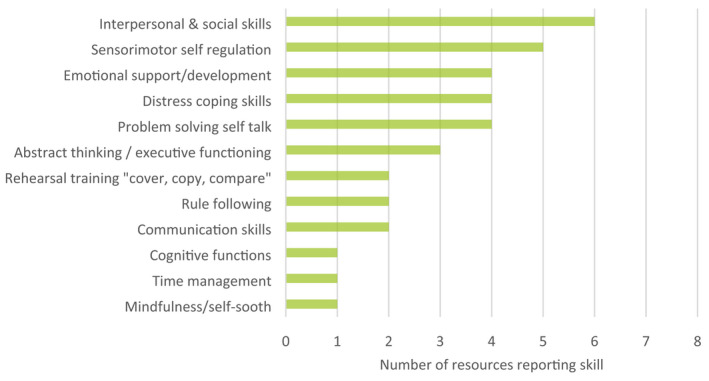
Evidence‐based skills that improve behavioural outcomes for students with FASD (total resources = 12)

Seventy‐five resources were identified, including 38 longer guides and books (32% peer‐reviewed), 22 short factsheets and booklets, eight peer‐reviewed literature reviews, three classroom planning tools and worksheets (one peer‐reviewed), two peer‐reviewed primary research studies, and two peer‐reviewed theses. Nine of 75 text‐based resources were current and had undergone peer review. These resources describe evidence‐based practices to support students with FASD in the school setting (Figures 3, 4). Short factsheets and booklets typically provide a list of tips for supporting students with FASD, although no reference to testing or evaluation is provided. Finally, current classroom planning tools provide educators with a framework for understanding the student's learning profile as well as mapping and tracking the use of accommodations to assist the student.

#### 3.4.2 Videos

Of the 26 videos identified, 22 were short videos or cartoons (ie, <20 min) and four were longer webinars or lectures. Webinars and lectures (n = 4) provided support strategies for behavioural management and academic success. Short videos and cartoons (n = 22) often focused on case studies and described strategies for a single domain, such as memory difficulties or sensory processing pace. No reference to evaluation or evidence for the effectiveness was provided for any of these resources.

#### 3.4.3 Podcasts

Ten podcast episodes from four podcast shows were identified. Episodes focused on classroom adjustments (to the environment, instructions and curriculum), establishing the educator‐student connection, tips from tutors and school interventions. No reference to scientific evidence or evaluation of support strategies was described in podcasts.

#### 3.4.4 Games

Two games for students were identified and one was of good quality. The *PAX Good Behaviour Game* introduces a positive classroom discipline system and is typically delivered across the first three years of primary school. A meta‐analysis of randomised controlled trials found the game has a small effect on improving classroom management and reducing behavioural problems.^25^

#### 3.4.5 Student interventions

Two evidence‐based intervention programs for students with FASD were identified. The *Math Interactive Learning Experience* program is a 6‐week intervention shown to significantly improve mathematics and handwriting skills and behavioural problems that interfere with learning readiness in children with FASD (Coles et al,^26^). The *Alert Program^®^* is an 8‐week program that significantly improves self‐regulation and executive functioning skills amongst students with FASD in some settings.^27^ A modified version was evaluated in remote Australian Aboriginal communities, however, there were no significant improvements to children's executive function or behaviour.^28^

#### 3.4.6 Professional development

Four current training modules were identified, developed in 2012‐2021. Two were developed based on published findings. The training module FASD, from the UK Department of Education, provides case studies on children with complex needs and includes common challenges for educators, how to respond and evidence‐based methods for optimising learning. Meanwhile, the e‐learning course, *FASD for School Staff*, developed by CanFASD, is available at a low cost and emphasises practical strategies for educators teaching students with FASD; the importance of educator‐caregiver collaboration; and provides instructional, environmental, and assessment strategies for the classroom. No reference to evaluation or evidence for the effectiveness was provided in any professional development resources.

### 3.5 Effective components

To identify current and effective strategies to improve support of students with FASD in the school setting, data were extracted from evaluated resources included in this review^5^, ^19^, ^21^, ^29^, ^30^, ^31^, ^32^, ^33^, ^34^, ^35^, ^36^, ^37^ and relevant peer‐reviewed studies that evaluated included resources published between 2000 and 2021.^12^, ^28^, ^38^ Figure 3 provides a thematic summary of evidence‐based educational strategies to support positive school experiences for students with FASD. Figure 4 summarises skills that should be targeted in students with FASD to improve behaviours and are supported by research evidence. Further details are provided in the Supplement.

### 3.6 Expert feedback

Of 40 experts invited to participate, 18 completed the online survey. Experts (94% female) were from Australia (n = 17) and the United Kingdom (n = 1) and worked as academics (n = 5), health professionals (n = 5), paediatricians (n = 3), in the not‐for‐profit sector (n = 1), social worker (n = 1), psychologist (n = 2) or as a school principal (n = 1). Years of experience in the field of FASD ranged from 3 to 30 years (M = 12.8; SD = 7.9).

Fourteen high‐quality resources were reviewed by experts and five (38%) were recommended for educators by all experts who reviewed them (Table S4). These included two short videos, one factsheet, one professional development and one podcast episode on support strategies. Seven resources (50%) received mixed feedback (either recommended or unsure) and two resources (14%) were not recommended by experts. Notably, most experts did not recommend any of the reviewed resources for the identification of FASD, as they were considered unsuitable for educators and did not utilise a strengths‐based approach.

Four key gap themes were identified from the expert survey: (1) low awareness about FASD, (2) low accessibility of current high‐quality resources, (3) need for evidence‐based referral resources and (4) need for evidence‐based resources providing classroom support strategies (see Supplement for further details).

## 4 DISCUSSION

### 4.1 Overview of resources

The aim of this scoping review was to identify resources that are publicly available to the primary/elementary education sector and provide staff with skills and strategies to improve the identification and support of students with indicators of FASD. In total, 124 relevant resources were identified and reviewed, 23 included information on identification, 119 included information to support students with FASD, but no resources provided information to facilitate referral. Resources were typically of average to good quality; however, less than 10% of resources had been formally evaluated. Most resources were developed in North America and made available to the education sector in the past eight years. Text‐based resources and videos were the most common type of resource identified, text‐based resources generally being higher quality than videos. Notably, many short format resources (ie, factsheets, booklets, <10 min videos)—which are arguably more accessible and usable for time‐poor educators—were of very poor to average quality. Consulted experts and educators noted high‐quality resources are hard to access due to both challenges in locating resources online and a lack of evidence‐based resources. Some experts called for a centralised Australian repository (website) of FASD resources for school communities including evidence‐based identification and support resources, available in short and accessible formats.

### 4.2 FASD identification and referral resources

This review identified only two evaluated screening tools available for use by educators^20^, ^22^ and two current, peer‐reviewed resources with information on FASD identification.^19^, ^21^ These tools are useful for screening neurobehavioural indicators prior to referral and diagnostic testing. However, the extent to which educators should be involved in screening children with suspected FASD is debated. Consulted experts consistently voiced the view that educators should not be expected to screen students with FASD. Instead, educators should be equipped with evidence‐based resources that allow them to recognise functional impairments consistent with FASD and understand how they may impact a student's school experience (Table 3). Awareness of functional impairments can assist educators in determining the strengths and weaknesses of a student with suspected FASD and enable educators to personalise the student's learning by incorporating evidence‐based support strategies (Figures 3, 4). In some circumstances, educators may seek to discuss referral pathways for clinical assessment with a student's caregiver. At present, there are no publicly available resources to facilitate these discussions, which were considered a strong need by all consulted experts. Additionally, we identified no high‐quality professional development resources providing information on identifying FASD‐related functional impairments. Development of an online FASD training module for educators on identification and referral may therefore be warranted.

### 4.3 FASD support resources

Thirty good to excellent quality support resources were current, including 17 guides and books, three booklets and factsheets, three professional development resources, two student interventions, two podcast episodes, one webinar, one short video and one game for students. Although there are a variety of high‐quality resources available to educators, experts note it is difficult for educators to locate these resources given they are spread across multiple websites and suggest the need for a centralised repository of high‐quality resources, such as FASD Hub or a dedicated platform for educators. Furthermore, many text‐based FASD resources identified are over 50 pages long and educators noted that time constraints are a key barrier to accessing resources. Development of short format resources, such as fact sheets or explainer videos that provide classroom strategies to support students with FASD (Figure 3) and skills to improve behavioural outcomes (Figure 4) are likely to be more useful and accessible for educators.

In alignment with findings from previous reviews of FASD interventions,^8^, ^9^, ^10^ we identified few intervention options for implementation by educators in schools. There is an urgent need for intervention trials that target both primary neurobehavioural consequences and secondary outcomes of FASD.

Finally, there is generally a lack of formal evaluation of resources and support strategies. The evidence base is often unclear, as reflected in the quality ratings. It is recommended that developers of future FASD support resources: (1) only include evidence‐based strategies, (2) test the utility and effectiveness of the resource with target users and (3) clearly state the evidence base of the resource. Adoption of these recommendations will improve the quality and utility of FASD support resources for educators.

### 4.4 Priority areas for resource development and research

This scoping review and consultation with experts and educators have identified five priority areas for future resource development and research: (1) development and evaluation of guides that facilitate discussions between educators and caregivers about referral pathways for clinical assessment, (2) development of short‐format resources that outline evidence‐based FASD support and accommodation strategies in an accessible format for educators, (3) development of strengths‐focused, school‐based FASD interventions and evaluation of such interventions by randomised trial, (4) evaluation of resources for feasibility and effectiveness and (5) improved access to high‐quality resources for the school community via online platforms, including professional development and training. Addressing these priorities will ensure that educators are better equipped with a suite of resources that allow them to effectively recognise and respond to students with or suspected of having FASD.

### 4.5 Limitations

First, the search strategy focused on websites and databases in Australia, New Zealand, Canada, the US and the UK. This strategy was employed as previous reviews show this is where most resources have been developed.^8^ Furthermore, the pre‐registered search strategy was restricted to FASD resources, despite the knowledge that students with FASD have a clinical presentation that overlaps with other neurodevelopmental disorders. However, a broader focus on resources for other neurodevelopmental disorders would have substantially widened the scope of the review and distracted from our primary and secondary research questions which are related to the availability of FASD‐specific resources. Additionally, a broader examination of FASD‐related literary and artistic work that can increase educator understanding could be explored in future research. Finally, there was a low participation rate from FASD experts, and this may be the result of a lengthy online survey. A shorter survey or focus groups may have been more engaging and increased participation rate.

### 4.6 Conclusions

This scoping review identified 124 publicly available resources for educators that provide information on the identification and support of primary/elementary school‐aged students with FASD. Most resources were of average to good quality but few were formally evaluated. Generally, short‐format, easy‐to‐access resources were of poor to average quality and less accessible guides and books were of higher quality. Following the review and consultation with experts and educators, priority areas for future resource development were identified, including development and evaluation of referral guides and evidence‐based short‐format resources and evaluation of interventions targeting primary and secondary consequences of FASD in students. Experts called for increased access to high‐quality resources for school communities including evidence‐based resources for the identification and support of students with FASD. Addressing these priority areas will better equip educators with resources that allow them to effectively identify and support students with FASD.

## CONFLICT OF INTEREST

None to declare.

## Supporting information

Supplementary Material
