# Supplementary material for: Fetal alcohol spectrum disorder resources for educators: A scoping review
Source: Health Promot J Austr. 2022 Jan 26;33(3):797–809. doi: 10.1002/hpja.574 (PMC9544813; doi:10.1002/hpja.574)
Supplement: Supplementary file 1 — Supplementary Material [file HPJA-33-797-s001.docx]

Supplement File.

**Methods**

Identifying relevant resources

Resources were identified by searching nine peer-reviewed databases (i.e., PsycINFO, PubMED, Scopus, Web of Science, ERIC, Cochrane Library, MEDLINE, EMBASE, CINAHL); 11 grey literature websites (Google advanced search [first 100 results per search], FASD Hub Australia, NOFASD Australia, HealthInfoNet, Australian Federal and State Department of Education websites, Australian Federal and State Department of Health websites, Education Services Australia, National Organisation for FASD [UK], The National Organization for Fetal Alcohol Syndrome [US], Children’s Healthcare Canada, The Provincial Outreach Program for Fetal Alcohol Spectrum Disorder); two app stores (Apple App Store, Google Play); two podcast streaming services (Apple Podcast, Spotify); and contacting 15 experts (five responded with resource suggestions). Experts included academics and health professionals with relevant experience regarding FASD or the education sector.

FASD-related search terms included “fetal alcohol spectrum disorder”, “fetal alcohol syndrome”, “alcohol related neurodevelopmental disorder”, “alcohol related birth defects”, “prenatal alcohol exposure”, and “alcohol use during pregnancy”. Resource-related terms included “program”, “intervention”, “management”, “guidelines”, “guide”, “policy”, “factsheet”, “information sheet”, “booklet”, “video”, “screening tool”, and “assessment”. Finally, setting-related terms included “school”, “school based”, “teacher”, and “educator”. Where possible, the same search terms and fields were used in database searches, using the websites’ search function. When the capacity of the website did not allow for multiple search terms, singular phrases were searched (e.g., “fetal alcohol spectrum disorder”). Searches for alternate spelling were also conducted (e.g., fetal vs. foetal). The search strategy used for Ovid databases (MEDLINE, EMBASE, PsycINFO, ERIC) is provided in Table S1.

Resources were included in the review if they were: 1) relevant for primary/elementary school educators (i.e., principals, teachers, education assistants, school psychologists, allied health teams and nurses, Indigenous Liaison Officers, or other school staff); 2) designed to build capacity among educators in identifying students with problems consistent with FASD, or supporting students with FASD; and 3) currently publicly available in English, including free and fee-based resources.

**Table S1.** Search strategy for Ovid databases (MEDLINE, ERICC, PsycINFO, EMBASE).

| 1 | (“fetal alcohol spectrum disorder” or “foetal alcohol spectrum disorder” or “fetal alcohol syndrome” or alcohol related neurodevelopmental disorder” or “alcohol related birth defects” or “prenatal alcohol exposure” or “alcohol use during pregnancy”).mp. [mp=ti, ab, hw, tn, ot, dm, mf, dv, kw, fx, dq, id, nm, kf, ox, px, rx, an, ui, sy, tc, tm, mh] |
| --- | --- |
| 2 | (school or “school based” or teacher or educator).mp. [mp=ti, ab, hw, tn, ot, dm, mf, dv, kw, fx, dq, id, nm, kf, ox, px, rx, an, ui, sy, tc, tm, mh] |
| 3 | 1 and 2 |
| 4 | (screen or “screening tool” or assessment).mp. [mp=ti, ab, hw, tn, ot, dm, mf, dv, kw, fx, dq, id, nm, kf, ox, px, rx, an, ui, sy, tc, tm, mh] |
| 5 | (program or intervention or management).mp. [mp=ti, ab, hw, tn, ot, dm, mf, dv, kw, fx, dq, id, nm, kf, ox, px, rx, an, ui, sy, tc, tm, mh] |
| 6 | (review or “systematic review” or “scoping review” or “meta-analysis”).mp. [mp=ti, ab, hw, tn, ot, dm, mf, dv, kw, fx, dq, id, nm, kf, ox, px, rx, an, ui, sy, tc, tm, mh] |
| 7 | (guide or guideline or guidelines or policy).mp. [mp=ti, ab, hw, tn, ot, dm, mf, dv, kw, fx, dq, id, nm, kf, ox, px, rx, an, ui, sy, tc, tm, mh] |
| 8 | (factsheet or booklet or “information sheet” or video or app or application or game or gaming or “virtual reality” or interactive or podcast).mp. [mp=ti, ab, hw, tn, ot, dm, mf, dv, kw, fx, dq, id, nm, kf, ox, px, rx, an, ui, sy, tc, tm, mh] |
| 9 | 4 or 5 or 6 or 7 or 8 |
| 10 | 3 and 9 |
| 11 | Limit 10 to English language |

**Table S2.** The quality appraisal tool

| ***Component*** | ***Excellent*** | ***Good*** | ***Satisfactory*** | ***Poor*** |
| --- | --- | --- | --- | --- |
| **Evidence base** | Formally evaluated and findings published | No formal evaluation; Developed on the basis of published findings OR some testing among end-users has been conducted | No reference to formal evidence or testing; developed by expert consensus | Developed on the basis of personal opinion only |
| **Impact and utility** | Covers a range of relevant issues comprehensively | Covers more than one relevant issue | Covers a single issue of high importance | Brief resource, restricted coverage of issue |
| **Generalisability** | Relevant to one or more of the target groups | Some information relevant for the target group |  | Not relevant for any of the target groups |
| **Applicability** | Directly applicable to the Australian context | Applicable to the Australian context with some caveats |  | Not applicable to an Australian context/unsure |
| **Availability** | Readily available at no cost | Available at low cost | Available at significant cost ($100+) | Not readily available |
| **Currency** | Resource is current / regularly updated | Resource is current but not updated regularly | Resource is not current but contains up-to-date information and terminology (≥10 years) | Resources contains out of date information and terminology |
| **Ease of Use** | Easy to use or navigate |  |  | Difficult to use or overly complex |
| **Credibility** | University-based or Government funded, reputable developers |  |  | Developers or organisation not reputable |

*Note.* Based on an adaption of the NHMRC Evidence Statement Form, the NHMRC Body of Evidence Matrix and the iCAHE Guidelines Quality Checklist.

**Results**

**Table S3.** List of resources identified in the scoping review

| **Resource** | | **Resource type** | **Year** | **Developer/Author** | **Quality** |
| --- | --- | --- | --- | --- | --- |
| **Identification resources** | | | | | |
| **Screening Tools** | | | | | |
| 1 | Neurobehavioural Screening Tool | Questionnaire (peer reviewed) | 2009 | Nash et al. | Excellent |
| 2 | FASCETS Neurobehavioral Screening Tool | Questionnaire | Unknown | FASCETS | Excellent |
| 3 | FASD: A Checklist | Questionnaire | Unknown | NOFASD | Average |
| 4 | The FAS Screen | Questionnaire (peer reviewed) | 1999 | Burd et al. | Average |
| 5 | Medicine Wheel Student Index | Questionnaire | 2000 | Elsipogtog First Nations community | Average |
| **Text-based** | | | | | |
| 6 | Fetal Alcohol Spectrum Disorder: Strategies for Learning, Behaviour and Communication | Guide | 2015 | Dr Barry Parsonson  Ministry of Education (NZ) | Excellent |
| 7 | Innovative educational interventions with school-aged children affected by fetal alcohol spectrum disorders (FASD) | Book chapter (peer reviewed) | 2011 | Coles et al. | Good |
| 8 | Fetal alcohol spectrum disorders: Understanding the effects of prenatal alcohol exposure and supporting students | Literature review (peer reviewed) | 2007 | Green | Good |
| 9 | Educating Students with FASD: Linking Policy, Research and Practice | Literature review; primary research study (peer reviewed) | 2017 | Millar et al. | Good |
| 10 | What Educators Need to Know about FASD: Working together to educate children in Manitoba with Fetal Alcohol Spectrum Disorder | Guide | 2018 | Healthy Child Manitoba  Manitoba Education and Training | Good |
| 11 | Understanding Fetal Alcohol Spectrum Disorders (FASD): A comprehensive guide for Pre-K-8 educators | Guide | 2008 | Dr Chandra Zieff, Dr Rochelle Schwarts-Bloom and Mark Williams Duke University | Good |
| 12 | Take Another Look: A Guide on Fetal Alcohol Spectrum Disorders for School Psychologists and Counselors | Guide | 2020 | NYS FASD Interagency Workgroup | Good |
| 13 | Understanding Fetal Alcohol Spectrum Disorders (FASD): What Educators need to know | Guide | 2019 | NHS Ayrshire and Arran | Good |
| 14 | Guided Growth: Educational and Behavioral Interventions for Children and Teens with FASD and Early Trauma | Book | 2020 | Ira J. Chasnoff & Ronald J. Powell | Good |
| 15 | What Early Childhood Educators Need to Know about Fetal Alcohol Spectrum Disorder (FASD) | Guide | 2010 | Healthy Child Manitoba  Manitoba Education and Training | Average |
| 16 | Making a Difference: Working with students who have Fetal Alcohol Spectrum Disorders | Guide | 2006 | Yukon Department of Education | Average |
| 17 | Fetal Alcohol Syndrome: Implications for Educators. | Guide (peer reviewed) | 1998 | Ackerman | Average |
| 18 | What Educators Need To Know about Having Students with Fetal Alcohol Syndrome and Fetal Alcohol Effects in the Classroom: Issues, Identification, Intervention & Instructional Strategies. | Guide (peer reviewed) | 1995 | Finlay & Sorenson | Average |
| 19 | Teaching Students with Fetal Alcohol Syndrome/Effects: A Learning Resource for Teachers | Guide | 1996 | British Columbia Ministry of Education | Poor |
| 20 | Awareness of Chronic Health Conditions: What the Teacher Needs To Know. | Guide (peer reviewed) | 1995 | British Columbia Ministry of Education | Poor |
| 21 | Recognizing and Managing Children with Fetal Alcohol Syndrome/Fetal Alcohol Effects: A Guidebook. | Book (peer reviewed) | 1997 | McCreight | Very poor |
| **Video** | | | | | |
| 22 | NOFASD Webinar for teachers and educators - August 2016 | Webinar | 2016 | Anne Heath  NOFASD | Good |
| **Professional Development** | | | | | |
| 23 | Supporting Students with Fetal Alcohol Spectrum Disorders | Learning/ training modules | 2013 | Edmonton Regional Learning Consortium University of Alberta Learning Network Educational Services | Average |
| **Support resources** | | | | | |
| **Text-based** | | | | | |
| 1 | FASD: From Isolation to Inclusion in Australian Schools | Thesis (peer reviewed) | 2016 | Dee Basaraba | Excellent |
| 2 | Fetal Alcohol Spectrum Disorder: Strategies for Learning, Behaviour and Communication | Guide | 2015 | Dr Barry Parsonson  Ministry of Education (NZ) | Excellent |
| 3 | Innovative educational interventions with school-aged children affected by fetal alcohol spectrum disorders (FASD) | Book chapter (peer reviewed) | 2011 | Coles et al. | Good |
| 4 | Guidelines for Fetal Alcohol Spectrum Disorder and Literacy and Learning | Guide (peer reviewed) | 2013 | Mitten | Good |
| 5 | Educating Children and Young People with Fetal Alcohol Spectrum Disorders: Constructing Personalised Pathways to Learning | Book | 2012 | Dr Carolyn Blackburn, Prof Barry Carpenter, Jo Egerton | Good |
| 6 | Guided Growth: Educational and Behavioral Interventions for Children and Teens with FASD and Early Trauma | Book | 2020 | Ira J. Chasnoff & Ronald J. Powell | Good |
| 7 | Explained by Brain: The FASD Workbook for Parents, Carers & Educators (including Reflections & Resources Booklet) | Book | 2020 | Dr Vanessa Spiller | Good |
| 8 | Trying Differently Rather Than Harder | Book | 1999 (revised 2016) | Diane Malbin, FASCETS | Good |
| 9 | Re: Defining Success. A Team Approach to Supporting Students with FASD | Guide | 2009 | Alberta Education | Good |
| 10 | Introduction to Teachers | Guide | Unknown | NOFASD | Good |
| 11 | What Educators Need to Know about FASD: Working together to educate children in Manitoba with Fetal Alcohol Spectrum Disorder | Guide | 2018 | Healthy Child Manitoba  Manitoba Education and Training | Good |
| 12 | Foetal Alcohol Spectrum Disorder: Focus on Strategies | Guide | 2009 | Dr Carolyn Blackburn  Sunfield Research Institute  Worcestershire’s Early Years and Childcare Service | Good |
| 13 | Understanding Fetal Alcohol Spectrum Disorders (FASD): A comprehensive guide for Pre-K-8 educators | Guide | 2008 | Dr Chandra Zieff, Dr Rochelle Schwarts-Bloom and Mark Williams Duke University | Good |
| 14 | Supporting School-age Children with Fetal Alcohol Spectrum Disorder | Guide | 2019 | Dr Amanda Wilkins and Dr Robyn Williams  Developmental Disability WA | Good |
| 15 | Teaching Students with Fetal Alcohol Spectrum Disorder: Building Strengths, Creating Hope | Guide | 2004 | Alberta Learning | Good |
| 16 | Understanding Fetal Alcohol Spectrum Disorders (FASD): What Educators need to know | Guide | 2019 | NHS Ayrshire and Arran | Good |
| 17 | Reach to Teach: Educating Elementary and Middle School Children with Fetal Alcohol Spectrum Disorders | Guide | 2007 | Substance Abuse and Mental Health Services Administration | Good |
| 18 | Take Another Look: A Guide on Fetal Alcohol Spectrum Disorders for School Psychologists and Counselors | Guide | 2020 | NYS FASD Interagency Workgroup | Good |
| 19 | Fetal alcohol spectrum disorder (FASD) and complex trauma: A resource for educators | Guide | 2018 | Sue Thomas and Jane Weston Marinwarntikura Women's Resource Centre | Good |
| 20 | Guide to FASD and learning | Guide | Unknown | Ministry of Education (NZ) | Good |
| 21 | Talking About Special Education: Volume 2: Talking About Fetal Alcohol Spectrum Disorder: An Information Handbook | Booklet | 2018 | First Nations Education Steering Committee and the First Nations Schools' Association of British Columbia | Good |
| 22 | FASD in the Classroom | Factsheet | Unknown | Telethon Kids Institute | Good |
| 23 | Teaching a Student with FASD | Factsheet | 2017 | National Organisation for FASD (UK) | Good |
| 24 | Fetal Alcohol Spectrum Disorders (FASD) | Factsheet | 2017 | Special Education Resource Unit  South Australia Department of Education | Good |
| 25 | Educating Students with FASD: Linking Policy, Research and Practice | Literature review; primary research study (peer reviewed) | 2017 | Millar et al. | Good |
| 26 | How teachers can manage attention span and activity level difficulties due to Foetal Alcohol Syndrome in the classroom: an occupational therapy approach | Literature review; primary research study (peer reviewed) | 2009 | Assink et al. | Good |
| 27 | FASD: What types of intervention and rehabilitation are useful? | Literature review (peer reviewed) | 2007 | Kalberg & Buckley | Good |
| 28 | Fetal alcohol spectrum disorders: Understanding the effects of prenatal alcohol exposure and supporting students | Literature review (peer reviewed) | 2007 | Green | Good |
| 29 | Educational Needs and Care of Children with FASD | Literature review (peer reviewed) | 2015 | Millians | Good |
| 30 | Shaping the future for children with foetal alcohol spectrum disorders | Literature review (peer reviewed) | 2018 | Blackburn et al. | Good |
| 31 | Effects of a Universal School-Based Mental Health Program on the Self-concept, Coping Skills, and Perceptions of Social Support of Students with Developmental Disabilities | Primary research study (peer reviewed) | 2020 | Katz et al. | Good |
| 32 | Exploring the New Zealand Child and Youth Profile as a collaborative tool to support educational planning for children with fetal alcohol spectrum disorder | Thesis (peer reviewed) | 2019 | Joanne van Wyk | Good |
| 33 | A Guide for Families and Communities | Book (peer reviewed) | 1997 | Streissguth | Average |
| 34 | The challenge of Fetal Alcohol Syndrome: Overcoming secondary disabilities | Book (peer reviewed) | 1997 | Streissguth et al. | Average |
| 35 | Intervention Strategies for School Age Children | Guide (peer reviewed) | 1990 | D’Entremont | Average |
| 36 | Fetal Alcohol Syndrome: Implications for Educators | Guide (peer reviewed) | 1998 | Ackerman | Average |
| 37 | What Educators Need To Know about Having Students with Fetal Alcohol Syndrome and Fetal Alcohol Effects in the Classroom: Issues, Identification, Intervention & Instructional Strategies. | Guide (peer reviewed) | 1995 | Finlay & Sorenson | Average |
| 38 | What Early Childhood Educators Need to Know about Fetal Alcohol Spectrum Disorder (FASD) | Guide | 2010 | Healthy Child Manitoba  Manitoba Education and Training | Average |
| 39 | Making a Difference: Working with students who have Fetal Alcohol Spectrum Disorders | Guide | 2006 | Yukon Department of Education | Average |
| 40 | Supporting and Teaching Learners with FASD - Educator Handout | Guide | 2019 | POPFASD | Average |
| 41 | Teaching Students with Fetal Alcohol Spectrum Disorders | Guide | 2005 | Florida Department of Education Bureau of Exceptional Education and Student Services | Average |
| 42 | Tips for Teachers | Guide | Unknown | FASD Network Saskatchewan | Average |
| 43 | Multi-model strategies for readers with FASD | Guide | Unknown | Linda Wason-Ellam | Average |
| 44 | Fetal Alcohol Spectrum Disorders: Education Strategies | Book | 2009 | Centre for Disabilities  University of South Dakota | Average |
| 45 | Fetal Alcohol Syndrome Handbook | Book | 2002 | Centre for Disabilities  University of South Dakota | Average |
| 46 | Communication: For Learners with FASD and Other Complex Learning Needs | Booklet | 2018 | POPFASD | Average |
| 47 | Environment: For Learners with FASD and other Complex Learning Needs | Booklet | 2019 | POPFASD | Average |
| 48 | Tailored teaching strategies for supporting children living with FASD and Universal and inclusive accommodations to create FASD sensitive environments | Factsheet | Unknown | Dr Sara McClean  Emerging Minds | Average |
| 49 | Brain not Blame | Factsheet | 2013 | Professionals with Parachutes | Average |
| 50 | FASD Educator | Factsheet | 2013 | Professionals with Parachutes | Average |
| 51 | Goal Setting Tip Sheet | Factsheet | 2013 | Professionals with Parachutes | Average |
| 52 | Foetal Alcohol Spectrum Disorder: Information Sheets | Factsheet | 2009 | Jo Egerton  Sunfield Research Institute  Worcestershire's Early Years and Childcare Service | Average |
| 53 | Common behaviours, misinterpretations, and characteristics of students with FASD | Factsheet | Unknown | Ministry of Education (NZ) | Average |
| 54 | FASD in Focus: Education Profile | Factsheet | 2020 | National Organisation for FASD (UK) | Average |
| 55 | FASD in Focus: Tips for Educators | Factsheet | 2020 | National Organisation for FASD (UK) | Average |
| 56 | FASD: What School Systems Should Know about Affected Students | Factsheet | 2014 | NOFAS (USA) | Average |
| 57 | Fetal Alcohol Spectrum Disorder Fact Sheet | Factsheet | 2014 | Minnesota Association for Children's Mental Health | Average |
| 58 | Eight Magic Keys | Factsheet | 2016 | POPFASD (based on Deb Evenson and Jan Lutke) | Average |
| 59 | Suggested behavioral interventions in the classroom to assist students prenatally exposed to drugs | Literature review (peer reviewed) | 1998 | McLaughlin et al. | Average |
| 60 | Toward Better Collaboration in the Education of Students with Fetal Alcohol Spectrum Disorders: Integrating the Voices of Teachers, Administrators, Caregivers, and Allied Professionals | Primary research study (peer reviewed) | 2013 | Job et al. | Average |
| 61 | LEIC Planning Tool | Planning tool | 2018 | POPFASD | Average |
| 62 | Effective Teaching for FAS & FAE Children | Guide (peer reviewed) | 1997 | Root | Poor |
| 63 | Awareness of Chronic Health Conditions: What the Teacher Needs To Know | Guide (peer reviewed) | 1995 | British Columbia Ministry of Education | Poor |
| 64 | Fantastic Antone Succeeds | Book | 1993 | Judith Kleinfeld, Siobhan Wescott | Poor |
| 65 | Teaching Students with Fetal Alcohol Syndrome/Effects: A Learning Resource for Teachers | Guide | 1996 | British Columbia Ministry of Education | Poor |
| 66 | The Kids in My Class II: Students with Low Incidence Special Needs in regular classrooms | Guide | 2008 | Delta School District (USA) | Poor |
| 67 | Hey Teacher | Factsheet | Unknown | Fetal Alcohol Network New Zealand | Poor |
| 68 | Fetal Alcohol Syndrome Information Sheet | Factsheet | Unknown | Minnesota Department of Education | Poor |
| 69 | Instructional Tips: Supporting the Educational Needs of Students with Fetal Alcohol Spectrum Disorders | Literature review; primary research study (peer reviewed) | 2006 | Ryan | Poor |
| 70 | INVEST in your Intervention Efforts | Planning tool/work sheet | 2013 | Professionals with Parachutes | Poor |
| 71 | Recognizing and Managing Children with Fetal Alcohol Syndrome/Fetal Alcohol Effects: A Guidebook. | Book (peer reviewed) | 1997 | McCreight | Very poor |
| 72 | A Sourcebook of Successful School-based Strategies for Fetal Alcohol and Drug-Affected Students | Guide (peer reviewed) | 1994 | Osborne | Very poor |
| 73 | Fetal Alcohol Syndrome Factsheet (for Schools) | Factsheet | 2015 | Kids Health | Very poor |
| 74 | Learning Landscapes: Supporting Sensory and Processing Behaviors associated with FASD | Factsheet | Unknown | Suzie Kuerschner | Very poor |
| 75 | Fetal Alcohol Syndrome: A Training Manual To Aid in Vocational Rehabilitation and Other Non-Medical Services | Planning tool/work sheet (peer reviewed) | 1999 | LaDue et al. | Very poor |
| **Video** | | | | | |
| 76 | NOFASD Webinar for teachers and educators - August 2016 | Webinar | 2016 | Anne Heath  NOFASD | Good |
| 77 | NOFAS Webinar: Students with FASD: Simple strategies for behavioural and academic success | Webinar | Unknown | NOFAS (USA) | Good |
| 78 | 8 Magic Keys: Strategies for Students with FASD | Short video (cartoon) | Unknown | Deb Evenson and Jan Lutke  NOFAS (USA) | Good |
| 79 | Strategies for improving outcomes | Short video | Reviewed 2015 | POPFASD | Good |
| 80 | NOFAS Webinar: Triumph: Educating Students with an FASD | Webinar | 2015 | NOFAS (USA) | Average |
| 81 | Behaviour management - How to support someone with FASD | Lecture | Unknown | National Organisation for FASD (UK) | Average |
| 82 | My Brain, Me and FASD | Short video (cartoon) | 2018 | National Organisation for FASD (UK) | Average |
| 83 | Cause and Effect/Impulsivity | Short video | Reviewed 2014 | POPFASD | Average |
| 84 | Classroom Routines | Short video | Reviewed 2013 | POPFASD | Average |
| 85 | Environment | Short video | Reviewed 2013 | POPFASD | Average |
| 86 | Impacts | Short video | Reviewed 2019 | POPFASD | Average |
| 87 | Memory Difficulties | Short video | Reviewed 2019 | POPFASD | Average |
| 88 | Relationship | Short video | Reviewed 2016 | POPFASD | Average |
| 89 | Slow Processing Pace | Short video | Reviewed 2013 | POPFASD | Average |
| 90 | Teaching Social Skills - Elementary | Short video | Reviewed 2011 | POPFASD | Average |
| 91 | School Experiences | Short video | Reviewed 2014 | POPFASD | Poor |
| 92 | 6 things educators and school staff should know about FASD | Short video | 2016 | Nate Sheets | Very poor |
| 93 | Students Like Me: Episode 1 | Short video | Unknown | Vida Health Communications | Very poor |
| 94 | Students Like Me: Episode 2 | Short video | Unknown | Vida Health Communications | Very poor |
| 95 | Students Like Me: Episode 3 | Short video | Unknown | Vida Health Communications | Very poor |
| 96 | Students Like Me: Episode 4 | Short video | Unknown | Vida Health Communications | Very poor |
| 97 | Students Like Me: Episode 5 | Short video | Unknown | Vida Health Communications | Very poor |
| 98 | Students Like Me: Episode 6 | Short video | Unknown | Vida Health Communications | Very poor |
| 99 | Students Like Me: Episode 7 | Short video | Unknown | Vida Health Communications | Very poor |
| 100 | Students Like Me: Episode 8 | Short video | Unknown | Vida Health Communications | Very poor |
| 101 | Students Like Me: Episode 9 | Short video | Unknown | Vida Health Communications | Very poor |
| **Podcast** | | | | | |
| 102 | Classroom adjustments: Fetal Alcohol Spectrum Disorder (FASD) | Podcast episode | 2019 | Nationally Consistent Collection of Data on School Students with Disability Australian Government Initiative | Good |
| 103 | FASD through a variety of lenses: Episode 3 | Podcast episode | Reviewed 2019 | POPFASD | Good |
| 104 | FASD Informed: Connection is essential | Podcast episode | 2020 | North Carolina FASD Informed | Average |
| 105 | PopFASD TalkED: Episode 6 | Podcast episode | Reviewed 2020 | POPFASD | Average |
| 106 | FASD through a variety of lenses: Episode 6 | Podcast episode | Reviewed 2019 | POPFASD | Average |
| 107 | FASD Informed: Episode Tips from a tutor | Podcast episode | 2020 | North Carolina FASD Informed | Average |
| 108 | PopFASD TalkED: Episode 4 | Podcast episode | Reviewed 2020 | POPFASD | Average |
| 109 | PopFASD TalkED: Episode 5 | Podcast episode | Reviewed 2020 | POPFASD | Average |
| 110 | FASD Elephant: Episode FASD School Intervention: Getting Back to Ready | Podcast episode | 2008 | Michael L Harris | Very poor |
| 111 | PopFASD TalkED: Episode 7 | Podcast episode | Reviewed 2020 | POPFASD | Very poor |
| **Games** | | | | | |
| 112 | The PAX Good Behaviour Game | Game | Original (1969)  PAX version (2016) | PAXIS Institute | Good |
| 113 | Medicine Wheel Difference Game Cards | Game | 2000 | Elsipogtog First Nations community | Very poor |
| **Professional Development** | | | | | |
| 114 | Fetal Alcohol Spectrum Disorder | Learning/ training modules | 2019 | WA Department of Education | Good |
| 115 | FASD for School Staff Level II: Practical Strategies for the School Environment | Learning/ training modules | 2021 | CanFASD Research Network | Good |
| 116 | Foetal Alcohol Spectrum Disorder | Learning/ training modules | 2012 | Complex Needs; UK Department of Education | Good |
| 117 | Supporting Students with Fetal Alcohol Spectrum Disorders | Learning/ training modules | 2013 | Edmonton Regional Learning Consortium University of Alberta Learning Network Educational Services | Average |
| **Programs/Interventions** | | | | | |
| 118 | The ALERT program | Program | 1990 | Mary Sue Williams and Sherry Shellenberger, TherapyWords, Inc. | Excellent |
| 119 | Math Interactive Learning Experience (MILE) | Program | 2007 | Kable et al.  Centre for Maternal Substance Abuse and Child Development, Emory University | Good |

*Classroom strategies to support students with FASD*

Figure 3 in the manuscript provides a thematic summary of evidence-based educational strategies to support positive school experiences for students with FASD. The most commonly reported strategies included 1) involvement and support from caregivers in education planning, 2) structured environments where the pace of tasks is matched to the child’s processing speed capacity, 3) clear and simple visuals to improve sensory processing and reduce distraction, 4) presentation of tasks in small steps with frequent, short, and specific instructions, and 5) a calm learning environment, which is free from clutter (i.e., decreased visual, auditory, and physical stimulation), has natural light, and provides dedicated calm spaces for time out (e.g., tent or enclosed space).

*Skill development to improve behavioural outcomes*

Figure 4 in the manuscript provides a thematic summary of skills that should be targeted in students with FASD to improve behaviours and are supported by research evidence. The most frequently reported skills required include 1) interpersonal and social skills (e.g., assertiveness, setting boundaries, conflict avoidance), 2) sensorimotor self-regulation strategies (e.g., impulse control: addressed by the *Alert Program®*), 3) emotional development (e.g., building self-concept), 4) mental health and coping skills for psychological distress, and 5) problem solving self-talk (e.g., *Math Interactive Learning Experience* program uses a three-stage model to encourage students to: focus on /plan how to address an important part of problem, act by verbalising the steps they will use to solve the problem, reflect on what they learned, and discuss what strategies helped them solve the problem). Furthermore, the importance of upskilling educators through professional learning was emphasised in some recent resources (Basaraba, 2016; van Wyk, 2019; Wagner et al., 2020). Professional developmental modules and educator training were recommended to increase knowledge and awareness of students with FASD and to ensure that evidence-based strategies for improving behavioural outcomes are implemented with fidelity.

**Table S4.** Resources reviewed by experts

| **Resource** | | **Resource category** | **Resource type** | **Year** | **Developer/Author** | **Quality** | **Expert** | | |
| --- | --- | --- | --- | --- | --- | --- | --- | --- | --- |
| **Screening Tools** | | | | | | | **Yes** | **No** | **Not sure** |
| 1 | Neurobehavioural Screening Tool | Identification | Screening tool | 2009 | Nash et al. | Excellent |  | n=2;  100% |  |
| 2 | FASCETS Neurobehavioral Screening Tool | Identification | Screening tool | Unknown | FASCETS | Excellent | n=1; 33% |  | n=2; 67% |
| **Text-based** | | | | | | |  |  |  |
| 3 | Fetal Alcohol Spectrum Disorder: Strategies for Learning, Behaviour and Communication | Identification, support | Literature review/guide | 2015 | Dr Barry Parsonson  Ministry of Education (NZ) | Excellent |  | n=2; 100% |  |
| 4 | Fetal alcohol spectrum disorder (FASD) and complex trauma: A resource for educators | Support | Guide | 2018 | Sue Thomas and Jane Weston Marinwarntikura Women's Resource Centre | Good | n=1; 50% |  | n=1; 50% |
| 5 | Talking About Special Education: Volume 2: Talking About Fetal Alcohol Spectrum Disorder: An Information Handbook | Support | Booklet | 2018 | First Nations Education Steering Committee and the First Nations Schools' Association of British Columbia | Good | n=1; 33% |  | n=2; 67% |
| 6 | Guide to FASD and learning | Support | Online guide | Unknown | Ministry of Education (NZ) | Good | n=4; 50% |  | n=4; 50% |
| 7 | FASD in the Classroom | Support | Factsheet | Unknown | Telethon Kids Institute | Good | n=2; 100% |  |  |
| 8 | Teaching a Student with FASD | Support | Factsheet | 2017 | National Organisation for FASD (UK) | Good | n=1;  33% |  | n=2; 67% |
| **Video** | | | | | | |  |  |  |
| 9 | 8 Magic Keys: Strategies for Students with FASD | Support | Cartoon | Unknown | Deb Evenson and Jan Lutke  NOFAS (USA) | Good | n=1; 100% |  |  |
| 10 | Strategies for improving outcomes | Support | Short video | Reviewed 2015 | POPFASD | Good | n=2;  100% |  |  |
| **Professional Development** | | | | | | |  |  |  |
| 11 | Fetal Alcohol Spectrum Disorder | Support | Professional development learning/ training modules | 2019 | WA Department of Education | Good | n=1; 100% |  |  |
| 12 | Supporting Students with Fetal Alcohol Spectrum Disorders | Identification, support | Professional development learning/ training modules | 2013 | Edmonton Regional Learning Consortium University of Alberta Learning Network Educational Services | Average | n=1;  50% |  | n=1; 50% |
| **Podcast** | | | | | | |  |  |  |
| 13 | Classroom adjustments: Fetal Alcohol Spectrum Disorder (FASD) | Support | Podcast episode | 2019 | Nationally Consistent Collection of Data on School Students with Disability Australian Government Initiative | Good | n=2; 100% |  |  |
| 14 | FASD through a variety of lenses: Episode 3 | Support | Podcast episode | Reviewed 2019 | POPFASD | Good |  |  | n=3; 100% |

*Expert Feedback*

Most resources were consistently rated somewhat or very useful for educators (*n*=11;79%), easy or very easy to understand (*n*=12;86%) and used mostly or very appropriate language (*n*=8;57%). Fewer resources were rated as mostly or very likely to be effective (*n*=5;36%), mostly or very aligned with the current evidence base (*n*=6;43%), mostly or very aligned with FASD guidelines (*n*=6;43%), mostly or very aligned with a strengths-based approach (*n*=5;36%), and covered the topic in sufficient depth (*n*=3;21%).

Four key gap themes were identified from the expert survey: 1) low awareness about FASD, 2) low accessibility of current high-quality resources, 3) need for evidence-based referral resources, and 4) need for evidence-based management resources providing classroom strategies.

Firstly, experts indicated that there is low general awareness in the education sector about FASD and that educator training is needed to provide an overview of the disorder. Increasing educators’ knowledge and understanding of FASD and its impacts on learning was repeatedly noted as a key area for action. Experts further noted that although high quality resources exist, it is difficult for educators to locate them given they are spread across websites according to who developed them. Additionally, accessibility of high-quality resources was thought to be limited by the long format of many resources, such as the identified guides and webinars. Educators are often time poor and require evidence-based resources that consider these time limitations. There was strong support for the need for referral guides. Experts noted that educators should not be diagnosing FASD but should be equipped with evidence-based resources that allow them to recognise functional impairments in students and then seek referral. Finally, experts indicated that educators should be equipped with accessible, evidence-based resources that provide classroom strategies to support students with FASD. Notably, 60% of experts indicated that future development of resources providing strategies to support learning is the top priority. Although many support resources were identified through this review, the majority provide tips and strategies without a clear evidence base.

*Educator feedback*

In total, 33 Australian educators (91% female) completed the online survey. Consultees were teachers (*n* = 21; 64%), education assistants (*n* = 5; 15%), principals (*n* = 2; 6%), school counsellors (*n* = 2; 6%), school psychologists (*n* = 1; 3%), or did not disclose their role (*n* = 2; 6%) and worked in metropolitan (*n* = 18; 55%), regional (*n* = 9; 27%), and remote schools (*n* = 6; 18%). Years of experience in the education sector ranged from 1-43 years (*M* = 14.4; *SD* = 11.8).

Overall, 18 of 33 educators (55%) had accessed resources about FASD to support their work. Educators accessed resources that provided information on recognising symptoms (*n* = 16), strategies to support learning (*n* = 14) and behaviour in the classroom (*n* = 9), FASD awareness (*n* = 8), and referral pathways (*n* = 3). Educators typically found these resources via Google (*n* = 16), Government websites (*n* = 5), and from the school administration (*n* = 3). Despite most educators accessing FASD resources, only four (12%) indicated they had ‘go-to’ sources for high-quality information on FASD and support. Further, when asked about barriers to obtaining information about FASD, most indicated a lack of available resources (*n* = 23; 70%), paucity of time (to search for, read, view, or listen to lengthy resources; *n* = 18; 55%), and competing priorities (*n* = 18; 55%). Notably, most educators said it was important that resources were evidence-based (*n* = 30; 91%), developed by a credible source (*n* = 26; 79%), and supported by the Government (*n* = 18; 55%) or head of school (*n* = 16; 48%). Finally, educators indicated that the three most important resources they need in the school setting are 1) symptom recognition tools, 2) information on the impact of FASD on behaviour, and 3) how FASD disrupts learning ability.

**Preferred Reporting Items for Systematic reviews and Meta-Analyses extension for Scoping Reviews (PRISMA-ScR) Checklist**

| **SECTION** | **ITEM** | **PRISMA-ScR CHECKLIST ITEM** | **REPORTED ON PAGE #** |
| --- | --- | --- | --- |
| **TITLE** | | | |
| Title | 1 | Identify the report as a scoping review. | 1 |
| **ABSTRACT** | | | |
| Structured summary | 2 | Provide a structured summary that includes (as applicable): background, objectives, eligibility criteria, sources of evidence, charting methods, results, and conclusions that relate to the review questions and objectives. | 3 |
| **INTRODUCTION** | | | |
| Rationale | 3 | Describe the rationale for the review in the context of what is already known. Explain why the review questions/objectives lend themselves to a scoping review approach. | 4 - 5 |
| Objectives | 4 | Provide an explicit statement of the questions and objectives being addressed with reference to their key elements (e.g., population or participants, concepts, and context) or other relevant key elements used to conceptualize the review questions and/or objectives. | 5 |
| **METHODS** | | | |
| Protocol and registration | 5 | Indicate whether a review protocol exists; state if and where it can be accessed (e.g., a Web address); and if available, provide registration information, including the registration number. | 5 |
| Eligibility criteria | 6 | Specify characteristics of the sources of evidence used as eligibility criteria (e.g., years considered, language, and publication status), and provide a rationale. | 7 |
| Information sources* | 7 | Describe all information sources in the search (e.g., databases with dates of coverage and contact with authors to identify additional sources), as well as the date the most recent search was executed. | 6 |
| Search | 8 | Present the full electronic search strategy for at least 1 database, including any limits used, such that it could be repeated. | 7 and Table S1 |
| Selection of sources of evidence† | 9 | State the process for selecting sources of evidence (i.e., screening and eligibility) included in the scoping review. | 7 - 8 |
| Data charting process‡ | 10 | Describe the methods of charting data from the included sources of evidence (e.g., calibrated forms or forms that have been tested by the team before their use, and whether data charting was done independently or in duplicate) and any processes for obtaining and confirming data from investigators. | 8 |
| Data items | 11 | List and define all variables for which data were sought and any assumptions and simplifications made. | 8 and Table 1 |
| Critical appraisal of individual sources of evidence§ | 12 | If done, provide a rationale for conducting a critical appraisal of included sources of evidence; describe the methods used and how this information was used in any data synthesis (if appropriate). | 8 |
| Synthesis of results | 13 | Describe the methods of handling and summarizing the data that were charted. | 8 |
| **RESULTS** | | | |
| Selection of sources of evidence | 14 | Give numbers of sources of evidence screened, assessed for eligibility, and included in the review, with reasons for exclusions at each stage, ideally using a flow diagram. | 9 and Figure 1 |
| Characteristics of sources of evidence | 15 | For each source of evidence, present characteristics for which data were charted and provide the citations. | 10 and Table 1 |
| Critical appraisal within sources of evidence | 16 | If done, present data on critical appraisal of included sources of evidence (see item 12). | 10 - 16, Table 2 and Figure 2 |
| Results of individual sources of evidence | 17 | For each included source of evidence, present the relevant data that were charted that relate to the review questions and objectives. | 10 – 16, 18 – 21 |
| Synthesis of results | 18 | Summarize and/or present the charting results as they relate to the review questions and objectives. | 10 – 16, 17 – 18, Table 3, Figure 3 and 4 |
| **DISCUSSION** | | | |
| Summary of evidence | 19 | Summarize the main results (including an overview of concepts, themes, and types of evidence available), link to the review questions and objectives, and consider the relevance to key groups. | 21 |
| Limitations | 20 | Discuss the limitations of the scoping review process. | 25 |
| Conclusions | 21 | Provide a general interpretation of the results with respect to the review questions and objectives, as well as potential implications and/or next steps. | 26 |
| **FUNDING** | | | |
| Funding | 22 | Describe sources of funding for the included sources of evidence, as well as sources of funding for the scoping review. Describe the role of the funders of the scoping review. | 2 |

JBI = Joanna Briggs Institute; PRISMA-ScR = Preferred Reporting Items for Systematic reviews and Meta-Analyses extension for Scoping Reviews.

* Where *sources of evidence* (see second footnote) are compiled from, such as bibliographic databases, social media platforms, and Web sites.

† A more inclusive/heterogeneous term used to account for the different types of evidence or data sources (e.g., quantitative and/or qualitative research, expert opinion, and policy documents) that may be eligible in a scoping review as opposed to only studies. This is not to be confused with *information sources* (see first footnote).

‡ The frameworks by Arksey and O’Malley (6) and Levac and colleagues (7) and the JBI guidance (4, 5) refer to the process of data extraction in a scoping review as data charting*.*

§ The process of systematically examining research evidence to assess its validity, results, and relevance before using it to inform a decision. This term is used for items 12 and 19 instead of "risk of bias" (which is more applicable to systematic reviews of interventions) to include and acknowledge the various sources of evidence that may be used in a scoping review (e.g., quantitative and/or qualitative research, expert opinion, and policy document).

*From:* Tricco AC, Lillie E, Zarin W, O'Brien KK, Colquhoun H, Levac D, et al. PRISMA Extension for Scoping Reviews (PRISMAScR): Checklist and Explanation. Ann Intern Med. 2018;169:467–473. [doi: 10.7326/M18-0850](http://annals.org/aim/fullarticle/2700389/prisma-extension-scoping-reviews-prisma-scr-checklist-explanation).
